# In Vitro Evaluation of Electrochemotherapy Combined with Sotorasib in Pancreatic Carcinoma Cell Lines Harboring Distinct *KRAS* Mutations

**DOI:** 10.3390/ijms26157165

**Published:** 2025-07-24

**Authors:** Tanja Jesenko, Masa Omerzel, Tina Zivic, Gregor Sersa, Maja Cemazar

**Affiliations:** 1 Department of Experimental Oncology, Institute of Oncology Ljubljana, SI-1000 Ljubljana, Slovenia; tjesenko@onko-i.si (T.J.); momerzel@onko-i.si (M.O.); zivic.tina@gmail.com (T.Z.); gsersa@onko-i.si (G.S.); 2 Faculty of Medicine, University of Ljubljana, SI-1000 Ljubljana, Slovenia; 3 Faculty of Health Sciences, University of Ljubljana, SI-1000 Ljubljana, Slovenia; 4 Faculty of Health Sciences, University of Primorska, SI-6310 Izola, Slovenia

**Keywords:** electrochemotherapy, sotorasib, cisplatin, bleomycin, pancreatic cancer

## Abstract

Pancreatic cancer is among the deadliest malignancies, with limited treatment options and poor prognosis. Novel strategies are therefore urgently needed. Sotorasib, a KRAS G12C-specific inhibitor, offers targeted treatment for a small subset of patients with this mutation. Electrochemotherapy (ECT), which enhances the cytotoxicity of chemotherapeutic agents through electroporation-induced membrane permeabilization, has shown promise in various tumor types, including deep-seated malignancies such as pancreatic cancer. Combining ECT with sotorasib may potentiate antitumor effects in KRAS G12C-mutated pancreatic cancer; however, preclinical data on such combinations are lacking. This proof-of-concept study evaluated the cytotoxic effects of ECT using bleomycin (BLM) or cisplatin (CDDP) in combination with sotorasib in *KRAS G12C*-mutated MIA PaCa-2 and *KRAS G12D*-mutated PANC-1 pancreatic cancer cell lines. ECT alone significantly reduced cell viability, particularly in MIA PaCa-2 cells, where electric pulses induced approximately 75% cell death. Combining ECT with sotorasib resulted in an additive effect on *KRAS G12C*-mutated MIA PaCa-2 cells, though no synergy was observed, likely due to the high intrinsic sensitivity to electric pulses. These results support the potential of combining physical and molecular therapies in a subset of pancreatic cancer patients and lay the groundwork for further in vivo studies to optimize treatment parameters and explore clinical translatability.

## 1. Introduction

Pancreatic cancer accounts for 2% of all cancers [1]. The most common malignant pancreatic tumor is ductal adenocarcinoma, which often begins in the head of the pancreas [1]. At the time of diagnosis, only up to 20% of the patients are eligible for surgery [2]. In about half of cases, distant metastases are already present [1,2]. The estimated survival is short due to the high mortality rate [1]. Pancreatic cancer is often diagnosed late, contributing to the fact that it is the fourth leading cause of cancer-related deaths. Pancreatic cancer is one of the most difficult malignancies to treat. Standard treatments are surgery, chemotherapy, and radiotherapy, but advances in targeted therapy and immunotherapy are opening new possibilities [3,4,5].

One of the most commonly mutated genes in pancreatic ductal adenocarcinoma is *KRAS* (the Kirsten rat sarcoma viral oncogene homolog), which occurs in over 90% of cases [6]. This gene encodes KRAS, a small GTPase located in the cell membrane, which regulates cell growth, differentiation, and survival [6]. In pancreatic cancer, activating mutations lock KRAS in its active state, leading to uncontrolled cell proliferation, survival, and tumor progression. In approximately 1–2% of patients, a G12C mutation is present in the *KRAS* gene [7]. Sotorasib (AMG 510), a small molecule-targeted therapeutic that selectively inhibits the KRAS protein with the G12C mutation [8], is already approved by the regulatory authorities for the treatment of patients with *KRAS G12C*-mutated non-small-cell lung cancer [9]. Sotorasib is a specific inhibitor of KRAS G12C due to the unique conformation of its allosteric site. KRAS G12C can maintain alternative interactions with its downstream effectors through an active cycle between the GDP-bound and GTP-bound states [10,11]. This difference enables KRAS G12C to be locked into an inactive (GDP-bound) conformation by covalently binding sotorasib to the cysteine residue at position 12, which is a specific feature not possible in any other *KRAS* mutation, including G12D [10]. The covalent binding of sotorasib inhibits the ability of KRAS to switch to the active GTP-bound state. Consequently, this inhibition leads to the disruption of downstream signaling pathways, leading to inhibition of cell growth and induction of apoptosis [8]. Sotorasib has been investigated in clinical trials for the treatment of *KRAS* G12C-mutated pancreatic cancer. The ongoing phase I/II CodeBreaK 100 clinical trial (ClinicalTrials.gov ID NCT03600883) is designed to investigate the safety, tolerability, and efficacy of sotorasib in patients with various solid tumors, including pancreatic cancer, harboring *KRAS* G12C mutations. Interim results from this study have demonstrated encouraging anticancer activity with manageable toxicity profiles [7,12]. These findings highlight the potential of sotorasib as a novel treatment option for a defined subgroup of patients with pancreatic cancer with otherwise limited treatment options.

Electrochemotherapy (ECT) also represents a novel, promising local therapy for pancreatic cancer. ECT is a locoregional, non-thermal ablative treatment that combines chemotherapeutic agents, such as bleomycin (BLM) or cisplatin (CDDP), with electric pulses that transiently increase cell membrane permeability, thereby enhancing intracellular drug uptake [13]. This approach potentiates the local effects of chemotherapy while minimizing systemic toxicity due to the use of reduced drug doses. ECT is well-established for the treatment of cutaneous tumors, especially for basal cell carcinoma and melanoma, where ECT is included in the ESMO guidelines, and has also demonstrated feasibility, safety, and efficacy in the management of deep-seated malignancies, including primary liver tumors and unresectable colorectal liver metastases [14,15]. In the context of pancreatic cancer, preclinical studies in porcine and rabbit models have shown that ECT can be applied safely without causing major complications such as acute pancreatitis or vascular injury [16,17]. Furthermore, initial clinical studies have confirmed its feasibility and safety in a palliative setting, supporting its potential application in the treatment of pancreatic tumors [18,19]. Our recent clinical study performed on patients undergoing pancreaticoduodenectomy for pancreatic head ductal adenocarcinoma demonstrated that a hybrid treatment approach combining surgery with an intraoperative ECT of the posterior resection surface is feasible and safe, supporting the potential of this approach to reduce local recurrence and improve outcomes in pancreatic cancer [20]. The efficacy of the approach could be increased by concomitant therapy with targeted drugs, such as sotorasib or sunitinib, which was already evaluated in combination with ECT in a preclinical study in pancreatic carcinoma and demonstrated the synergistic effect of the treatments [21]. The combinations of ECT with other targeted drugs such as vemurafenib in melanoma or olaparib in breast cancer have already been reported, both demonstrating the synergistic effects of the treatments [22,23].

Given the aggressive nature of pancreatic carcinoma and the limited effectiveness of current treatments, exploring combination therapies that integrate local treatment modalities like ECT with systemic targeted agents such as sotorasib could potentially enhance therapeutic outcomes. Therefore, the present study was conducted as a proof-of-principle in vitro investigation to assess the potential of combining ECT with sotorasib in the treatment of pancreatic carcinoma with different KRAS mutation statuses. The aim of this study was to determine the cytotoxicity of ECT using BLM or CDDP and to evaluate the interactions of ECT with the targeted drug sotorasib in *KRAS* G12C-mutated MIA PaCa-2 and *KRAS* G12D-mutated PANC-1 pancreatic cancer cell lines [24].

## 2. Results

### 2.1. Selective Cytotoxicity of Sotorasib in KRAS G12C-Mutated Pancreatic Carcinoma Cell Line

**Figure 1:**
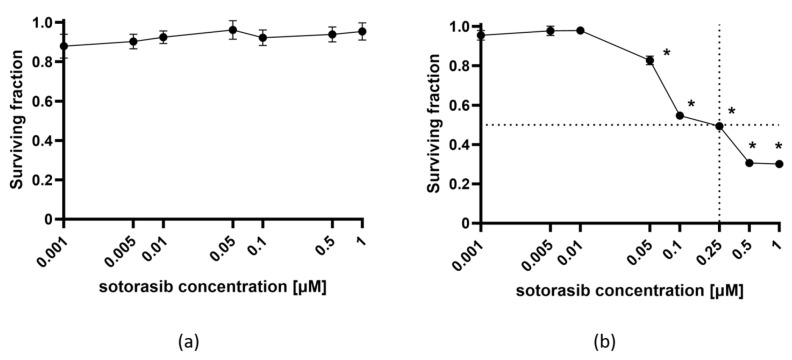
The cytotoxicity of sotorasib in PANC-1 (**a**) and MIA PaCa-2 (**b**) cell lines. The values are presented as the AM ± SEM. Dashed line represents IC_50_. * *p* < 0.05 vs. untreated control cells, n = 3.

To confirm the targeted action of sotorasib, the cytotoxicity of different concentrations was evaluated in PANC-1 and MIA PaCa-2 cell lines (Figure 1). As expected, sotorasib was not cytotoxic in PANC-1 cells (*KRAS* G12D) at any of the tested concentrations (Figure 1a). In contrast, the MIA PaCa-2 cell line (*KRAS* G12C) was very sensitive to sotorasib. Already, at a concentration of 0.05 µM, sotorasib significantly decreased the viability of cells to 80%. Higher concentrations further decreased the surviving fraction of MIA PaCa-2 cells (Figure 1b). The IC_50_ dose of sotorasib was determined at 0.25 µM. The experiments confirmed the selective toxicity of sotorasib in *KRAS* G12C-mutated cells.

### 2.2. Potentiation of Cytotoxic Drug Effectiveness by ECT and Combined Therapy

**Figure 2:**
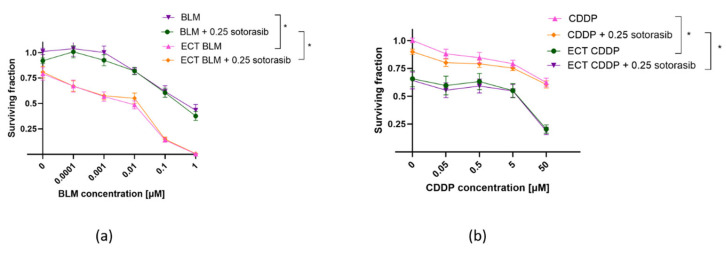
Surviving fraction of PANC-1 cells treated by ECT alone or combined with IC_50_ concentration (0.25 µM) of sotorasib. Treatment with BLM ECT (**a**) or with CDDP ECT (**b**). Values are presented as AM ± SEM. * *p* < 0.05 in BLM/CDDP vs. ECT BLM/CDDP or BLM/CDDP + 0.25 sotorasib vs. ECT BLM/CDDP + 0.25 sotorasib, n = 3. CDDP: cisplatin; BLM: bleomycin; ECT: electrochemotherapy.

**Figure 3:**
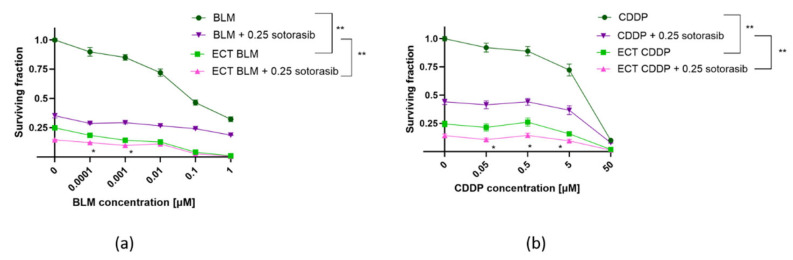
Surviving fraction of MIA PaCa-2 cells treated by ECT alone or combined with IC_50_ concentration (0.25 µM) of sotorasib. Treatment with BLM (**a**) or ECT with CDDP (**b**). Values are presented as AM ± SEM. * *p* < 0.05 in ECT BLM/CDDP vs. ECT BLM/CDDP + 0.25 sotorasib, ** *p* < 0.05 in BLM/CDDP vs. ECT BLM/CDDP and BLM/CDDP + 0.25 sotorasib vs. ECT BLM/CDDP + 0.25 sotorasib, n = 3. CDDP: cisplatin; BLM: bleomycin; ECT: electrochemotherapy.

ECT was effective in both cell lines, regardless of mutational status (Figure 2 and Figure 3). MIA PaCa-2 cells were more sensitive to BLM alone compared to PANC-1 cells; however, this was not significant (Figure 2a and Figure 3a). ECT with BLM was significantly more cytotoxic in MIA PaCa-2 cells compared to PANC-1 cells (Figure 2a and Figure 3a). Similar effects were observed when CDDP was used as a cytotoxic drug in ECT; MIA PaCa-2 cells were again significantly more sensitive to CDDP than PANC-1 cells (Figure 2b and Figure 3b). However, there was no significant difference in the cell surviving fraction among the two tested cell lines when cells were treated with CDDP alone; both cell lines were resistant to CDDP. The difference was significant only at a 50 µM concentration of CDDP (Figure 2b and Figure 3b).

Electric pulses alone caused a significant reduction in the surviving fraction of MIA PaCa-2 cells (Figure 3). As this effect can be related to the cell size, the cell size in suspension was evaluated by the CytoSmart automated cell counter and analyzed by AxIS Vue software version 33.12, demonstrating that PANC-1 cells are significantly bigger compared to MIA PaCa-2 cells (Figure S1).

In PANC-1 cells, combined ECT and sotorasib treatment did not additionally reduce cell survival, regardless of the cytotoxic drug (BLM or CDDP) used for ECT (Figure 2). Such results were expected, since sotorasib alone did not have any effect on PANC-1 cells. The surviving fraction curve of combined therapy aligned with the ECT-only surviving fraction curve for BLM (Figure 2a) and CDDP (Figure 2b) ECT.

On the other hand, the surviving fraction of MIA PaCa-2 cells was further reduced when ECT was combined with sotorasib (Figure 3). The difference in cell survival among the ECT BLM vs ECT BLM + 0.25 sotorasib was significant only up to 0.001 µM of BLM (Figure 3a). In the case of CDDP, the ECT CDDP + 0.25 sotorasib combination was significantly more cytotoxic than ECT CDDP alone at concentrations up to 5 μM (Figure 3b).

The interactions between the two drug combinations were evaluated relative to the individual therapies. A significant increase in cytotoxicity was observed for the BLM ECT + 0.25 sotorasib combination compared to either treatment alone in both BLM concentrations: 0.0001 and 0.001 µM (Figure 3a). The calculated values were indicative of an additive effect based on Q and 2SE calculations (Table 1). A significant increase in the cytotoxicity of the combined ECT CDDP + 0.25 sotorasib treatment was observed at three CDDP concentrations: 0.05, 0.5, and 5 µM (Figure 3b). The effect was additive in all three CDDP concentrations based on Q and 2SE calculations (Table 1).

## 3. Discussion

Pancreatic carcinoma remains one of the deadliest cancers, with limited effective treatment options and poor prognosis. The *KRAS* G12C mutation represents a promising therapeutic target, and sotorasib, a novel inhibitor, is entering clinical use for these patients. This study demonstrates how the combination of ECT with targeted therapy, sotorasib may offer a new strategy to improve treatment efficacy.

ECT with BLM or CDDP caused a small decrease in viability compared to both electroporation alone and treatment without electroporation. The treatment of cells with BLM gradually reduced the viability of cells, reaching approximately 40% survival at the highest concentrations of BLM used. ECT further decreased cell survival in a dose-dependent manner; however, the decrease in cell survival was not highly potentiated at lower doses of BLM. Rather, the survival slowly decreased with the increasing concentration of BLM. CDDP alone had an even lower cytotoxic effect on both cell lines. Both cell lines showed treatment resistance to CDDP, which was also evident after ECT, with the increased cytotoxic effect of ECT being probably connected to the cytotoxicity of electric pulses up to the highest concentration of CDDP used. These results indicate that both pancreatic cell lines are quite resistant to BLM and CDDP treatment, with ECT increasing the cytotoxic effect of both chemotherapeutic drugs. Pancreatic carcinoma is known to display remarkable resistance to all treatment strategies, including chemotherapy, with multifactorial mechanisms contributing to chemotherapy resistance [25]. A remarkable treatment resistance to CDDP was already demonstrated in PANC-1 cells, while the cytotoxic effect was higher in MIA PaCa-2 cells, similar to that observed in our study [26]. A slow decrease in cell survival after increasing the concentration of BLM or BLM ECT was also reported for the PANC-1 cell line [27].

In the current study, the combined effects of ECT and sotorasib were additive rather than synergistic. One of the possibilities for this effect is the high intrinsic cytotoxicity of electric pulses alone, which resulted in an approximately 75% reduction in cell survival in the MIA PaCa-2 cell line. Given such a substantial cytotoxic effect from electric pulses alone, the potential for further enhancement by the addition of a cytostatic drug or sotorasib was inherently limited. However, as there was still 25% percent left to reach 0% survival, sotorasib could have had an even stronger effect, which may argue against the notion of maximal cytotoxic saturation. To address this, future studies could explore the use of modified electric pulse parameters to reduce the standalone cytotoxicity of ECT and better evaluate potential synergistic effects with sotorasib. However, it must be acknowledged that such pulse parameters diverge from those currently applied in clinical ECT protocols [15], potentially limiting the translational relevance of these findings. Another explanation for the additive effect could be different mechanisms of action of ECT and sotorasib. ECT induces a fast cell death usually via necrosis and apoptosis due to high concentration of cytostatic drugs that damage the DNA [21,28]. The application of electric pulses also leads to some irreversible damage, leading to short- and long-term cell death [29,30]. On the other side, sotorasib is a targeted drug that inhibits a signaling pathway that mediates cell proliferation and does not cause lethal changes in the macromolecules [8]. Therefore, it could be anticipated that sotorasib acts on the cells that survive ECT treatment by mainly slowing down their proliferation, resulting in additive effects.

A 75% reduction in MIA PaCa-2 cell viability was observed three days after exposure to electric pulses at an electric field strength of 1300 V/cm. Previous reports have shown that similar pulse parameters (1300 V/cm) resulted in an approximately 50% decrease in MIA PaCa-2 cell survival when assessed one day post-treatment, suggesting a progressive cytotoxic effect over time [31]. At a lower field strength of 500 V/cm, cell viability remained relatively high (~80%) [31]; however, membrane permeabilization efficiency was below 50% [17], indicating suboptimal electroporation under these conditions. These findings highlight the high sensitivity of MIA PaCa-2 cells to electric pulse-induced cytotoxicity and underscore the difficulty of identifying pulse parameters that achieve effective membrane permeabilization while maintaining sufficient cell viability.

The pronounced cell death following pulse delivery is primarily attributed to irreversible electroporation. This phenomenon occurs when electroporated membranes fail to reseal, resulting in sustained membrane disruption. Such damage compromises cellular integrity and disrupts homeostatic regulation, leading to unregulated ion movement, the depletion of intracellular adenosine triphosphate (ATP) stores, and the accumulation of reactive oxygen species [30]. These stressors collectively exceed the capacity of cellular repair mechanisms and initiate cell death pathways. The nature and extent of cell death are highly dependent on the electroporation parameters, particularly the intensity, duration, and number of pulses [30]. The irreversible permeabilization is particularly observed in bigger cells that appear to be more fragile [32]. It was also postulated that bigger cells are permeabilized at lower field intensities than smaller ones; therefore, bigger cells are more sensitive to the lethal effect of the electric field [29]. This is not in line with our results of PANC-1 and MIA PaCa-2 cell size, demonstrating a significant difference between cell lines with PANC-1, which are around twice the size when compared to MIA PaCa-2 cells. In our study evaluating various tumor cell lines, the cell size was not correlated to electrosensitivity [33], indicating the importance of other mechanisms for the increased sensitivity of MIA PaCa-2 to electric pulses.

The pronounced cytotoxicity of electric pulses alone in the MIA PaCa-2 cell line limited the dynamic range for evaluating potential synergistic effects with sotorasib, thus representing one limitation of this study. Second, this study was limited to a single *KRAS* G12C-mutated pancreatic cancer cell line: MIA PaCa-2. Although this line is a relevant model for evaluating the effects of sotorasib, future studies involving additional *KRAS* G12C mutant cell lines and tumor models are necessary to confirm the reproducibility and broader applicability of these findings.

In conclusion, this proof-of-principle study demonstrates that the combination of ECT and sotorasib results in greater cytotoxicity than either treatment alone in vitro, with the overall additive nature of this effect. These findings indicate that ECT can increase the antitumor activity of targeted KRAS G12C inhibition, supporting the rationale for combining physical and molecular treatment modalities. The study provides a foundation for further investigation into animal tumor models to evaluate their therapeutic potential, optimize treatment parameters, and assess the safety and translational feasibility of this combinatorial approach.

## 4. Materials and Methods

### 4.1. Cell Lines

Two different human pancreatic cell lines, PANC-1 (CRL-1469) and MIA PaCa-2 (CRL-1420) (American Type Culture Collection, Manassas, VA, USA), were cultured in Advanced Dulbecco’s Modified Eagle Medium (DMEM; Gibco, Thermo Fisher Scientific, Waltham, MA, USA), supplemented with 5% (*v*/*v*) fetal bovine serum (FBS; Gibco), 10 mL/L L-glutamine (GlutaMAX; Gibco) and 1% (*v*/*v*) penicillin-streptomycin (stock solution, 10,000 U/mL, Gibco). For MIA, PaCa-2 media were supplemented with horse serum at a final concentration of 2.5% (*v*/*v*). PANC-1 was maintained at 37 °C in a 10% CO_2_ humidified incubator due to its higher sensitivity to pH deviations, whereas MIA PaCa-2 cells, bearing G12C mutation, were maintained at 5% CO_2_. Cells were routinely tested and confirmed to be free from mycoplasma infection using the MycoAlert^TM^ PLUS Mycoplasma Detection Kit (Lonza Group Ltd., Basel, Switzerland). Cell number was determined using the CytoSMART automatic cell counter (Axion Biosystems, Atlanta, GA, USA) and AxIS Vue software version 33.12 (Axion Biosystems), and cell size was determined using the “Cell size” function during the counting.

### 4.2. Drugs

A stock solution of 15000 IU BLM (Bleomycin medac, Medac, Wedel, Germany) was diluted in 0.9% NaCl saline (B. Braun Melsungen AG, 3632563, Melsungen, Germany) to produce five working solutions: 5, 0.5, 0.05, 0.005, and 0.0005 µM.

CDDP Kabi 1 mg/mL (Fresenius Kabi, Bad Homburg, Germany) was also diluted in 0.9% NaCl saline to five working solutions: 250, 25, 2.5, and 0.25 µM.

Sotorasib (AMG 510, 10 mM, Selleckchem, S7781, Houston, TX, USA) was diluted in 0.9% NaCl saline to 7 working solutions: 10, 5, 1, 0.5, 0.1, 0.05, and 0.01 µM.

### 4.3. Sotorasib Treatment

The monolayer of 80% confluent cells was trypsinized, centrifuged, and counted. Each well of a 96-well plate, 2 × 10^3^ PANC-1 cells and 4 × 10^3^ MIA PaCa-2 cells, was seeded in 90 µL of cell culture medium. In total, 10 µL of sotorasib was added to the cells to obtain final concentrations of 1, 0.5, 0.1, 0.05, 0.01, 0.005, and 0.0001 µM and these were incubated for 3 days. Then, the cell survival assay was performed as described below. A 3-day incubation interval was selected based on the doubling times of the cell lines and our laboratory’s standard operating procedure, which, together, ensured sufficient time to observe cytotoxic effects while minimizing variables such as the need for media replacement in longer assays with the addition of substances into the cell medium [22,34].

### 4.4. Cell Survival

Ten microliters of Presto Blue^®^ reagent (Thermo Fisher Scientific) was added to the wells and fluorescence intensity was measured one hour later using the Cytation 1 Multimodal Reader (BioTek Instruments, Winooski, VT, USA). The viability of the cells was normalized to untreated control cells (Ctrl).

### 4.5. ECT and Combined Treatment of ECT and Sotorasib

Cells were collected, centrifuged, and then resuspended in 1× Hanks’ Balanced Salt Solution (HBSS, Gibco), without FBS, which served as the pulsing medium. The final concentration was 2.5 × 10^7^ cells/mL. The mixture of a 10 µL BLM or CDDP solution of different concentrations and 40 µL cell suspension (1 × 10^6^ cells) was prepared for electroporation. In total, 50 µL of the mixture was pipetted between two stainless steel electrodes (with 2 mm gap), and electric pulses were delivered using an Electro Cell B10 electric pulse generator (LEROY Biotech, Saint-Orens-de-Gameville, France). The pulsing setup, commonly used for ECT, was selected, comprised 8 square-waved pulses, 1300 V/cm (260 V for 2 mm electrodes used in our experiment) and a 100 µs pulse duration at a frequency of 1 Hz. For all control samples, we also performed 0 V exposure and sham controls. The final concentrations of cytotoxic drugs were 5 times lower than working concentrations. Cells were then collected with a pipette and transferred into the well of a 24-well ultra-low attachment plate (Corning, 3473, Corning, NY, USA), and five minutes after pulse delivery, 1 mL of the cell culture medium was added. Afterwards, we transferred 2.1 µL (the calculated number for 2 × 10^3^ of PANC-1 cells) or 4.2 µL (the calculated number for 4 × 10^3^ of MIA PaCa-2 cells) from the ultra-low attachment plate to 87.9 µL or 85.8 µL of the cell culture medium (90 µL of the cumulative volume of cells and media) before adding 10 µL of the vehicle control (saline) and seeding the cells to 96-well plates and incubating for 3 days until the measurement of cell survival. Further experiments with combined therapy were performed with an IC_50_ dose of sotorasib. ECT was performed the same as described above. Afterwards, the cells were seeded as described above and 10 µL of sotorasib was added to the wells to reach a final 0.25 µM concentration. The same amount of vehicle control (saline) was added to the wells of groups that did not contain sotorasib. The cells were incubated for 3 days until the cell survival was measured. The medium was not replaced or supplemented during these 3 days in order to ensure the same concentration of sotorasib throughout the entire 3 days.

### 4.6. Statistical Analysis

A comparison between the two groups was performed using Student’s unpaired two-tailed *t*-test. The comparison of the means of more than two groups was statistically evaluated using one-way analysis of variance (one-way ANOVA) followed by Dunnett’s or Tukey’s multiple comparisons test. A *p*-value of <0.05 was considered to be statistically significant. The values in this study are presented as the mean (AM) ± standard error of the mean (SE). A sample size (n) for each experiment represents biological replicates; each replicate consists of an independent experiment with six technical replicates and is presented in each figure legend. For statistical analysis and the preparation of graphs, GraphPad Prism 10.4.1 (La Jolla, CA, USA) was used.

The combined effects of ECT and sotorasib treatment, including additivity, synergism, and antagonism, were evaluated. An additive effect occurs when the combined effect of two drugs equals the sum of their individual effects. Synergism is defined when the combined effect of two drugs exceeds the sum of their individual effects. These effects were calculated using the following formulas [35]:

(1) $$Q=\ln\left(\bar{x_{1}}\right)+\ln\left(\bar{x_{2}}\right)-\ln\left(\bar{x_{1+2}}\right)-\ln\left(\bar{x_{control}}\right)$$

(2) $$SE=\sqrt{\frac{{\left(\frac{\sigma_{1}}{\bar{x_{1}}}\right)}^{2}+{\left(\frac{\sigma_{2}}{\bar{x_{2}}}\right)}^{2}+{\left(\frac{\sigma_{1+2}}{\bar{x_{1+2}}}\right)}^{2}+{\left(\frac{\sigma_{control}}{\bar{x_{control}}}\right)}^{2}}{n_{1}+n_{2}+n_{1+2}+n_{control}}}$$

where $\bar{x}$ = average, n = the number of samples, σ = standard deviation, x_control_ = BLM or CDDP, x_1_ = ECT BLM or ECT CDDP, x_2_ = sotorasib 0.25 µM, and x_1+2_ = ECT BLM/CDDP + sotorasib. The results of both formulas provide the following information about the combined effects: Q < −2SE antagonism, −2SE < Q < 2SE additivity, and Q > 2SE synergism.

The combined effects of ECT and sotorasib treatment were calculated at three different concentrations of BLM and CDDP.

## Figures and Tables

**Table 1:** Calculated Q and 2SE values corresponding to treatment combination interactions.

Title 1	Q	2SE	Effect
ECT BLM 0.0001 + 0.25 sotorasib	−0.107	0.12	additive
ECT BLM 0.001 + 0.25 sotorasib	−0.062	0.08	additive
ECT BLM 0.01 + 0.25 sotorasib	−0.15	0.09	additive
ECT CDDP 0.05 + 0.25 sotorasib	0.12	0.23	additive
ECT CDDP 0.5 + 0.25 sotorasib	0.06	0.21	additive
ECT CDDP 5 + 0.25 sotorasib	0.19	0.21	additive

Q = calculated average values, SE = calculated standard error.

## Data Availability

The data presented in this study are available on request from the corresponding author.

## Supplementary Materials

The supporting information can be downloaded at: https://www.mdpi.com/article/10.3390/ijms26157165/s1.

## Abbreviations

The following abbreviations are used in this manuscript:

BLM	bleomycin
CDDP	cisplatin
ECT	electrochemotherapy
KRAS	Kirsten rat sarcoma viral oncogene homolog
