# Supplementary material for: In Vitro Evaluation of Electrochemotherapy Combined with Sotorasib in Pancreatic Carcinoma Cell Lines Harboring Distinct KRAS Mutations
Source: Int J Mol Sci. 2025 Jul 24;26(15):7165. doi: 10.3390/ijms26157165 (PMC12346384; doi:10.3390/ijms26157165)
Supplement: Supplementary file 1 [file ijms-26-07165-s001.zip › ijms-3701937-supplementary.pdf]

Supplementary Material

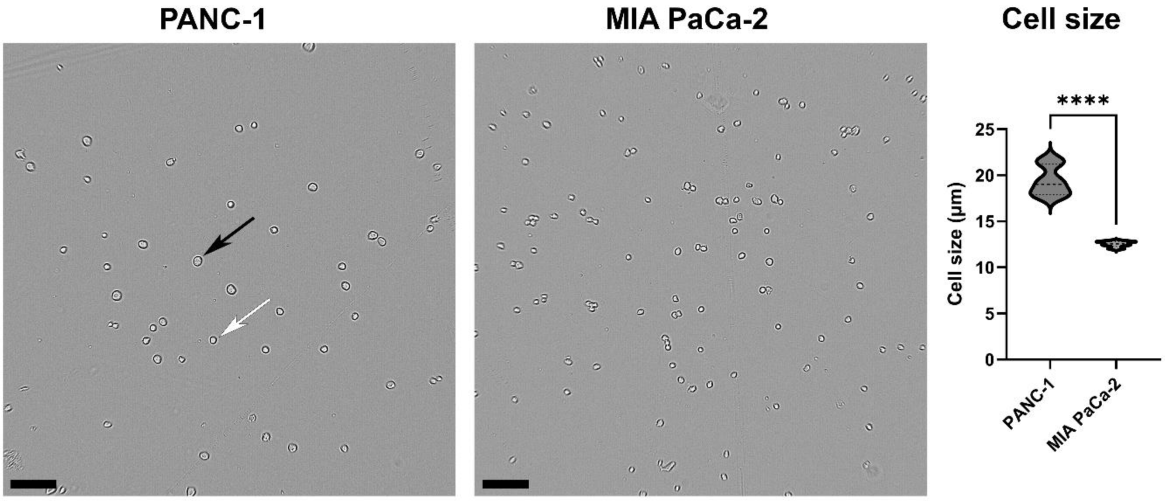

Figure S1. Representative images of PANC-1 and MIA PaCa-2 cells regarding the suspension and distribution of their measured sizes using the CytoSMART automatic cell counter and AxIS Vue software. Two subpopulations of PANC-1 cells were detected based on their size with the black arrow representing the larger subpopulation and the white arrow representing the smaller subpopulation. Scale bar: 100 μm. **** $p < 0.05$.
